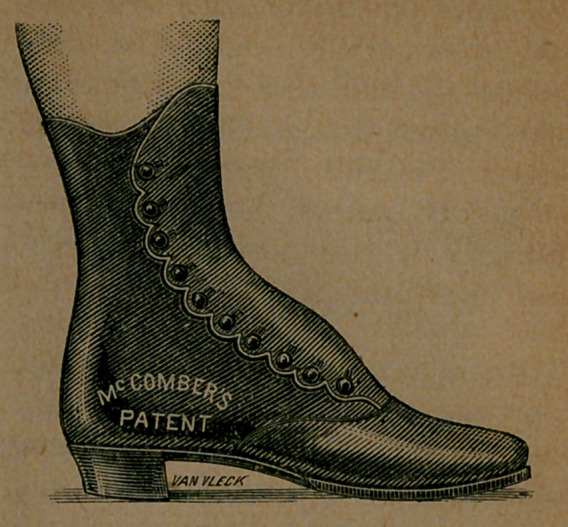# Deformed Feet

**Published:** 1875-03

**Authors:** 


### DEFORMED FEET.

We hope none of our readers will suppose that we have given up the feet of creation to rack and ruin and deformity, because we have been silent concerning the subject for a month or two. By no means. In this matter we have enlisted for the war; and on the identical line which we have heretofore laid down, we propose to fight it out.

But there are reasons why we have dropped the subject for a time. The fact is, we have been urged to keep silent; and the request has come from the inventor of the system, Joel McComber. It is not that he has lost confidence in it; it is not that he wants to study over it and perfect it; on the contrary, he was never more enthusiastic than now; never more fully convinced that his method of constructing lasts and boots and shoes, is the only true one.

But the fact is, we have attracted so much attention to the improved method, by our articles, and those who are wearing the McComber goods are so loud in their commendation of them, that the business has grown to rather formidable proportions,—somewhat beyond, in fact, the capacity of one man to manage. So we consented to hold our peace for a time; but only on condition that we be allowed to resume our warfare upon the abominable, distorting system of shoemaking in common use, at an early day. Mr. McComber now absolves us from our reluctant and temporary pledge of silence, and tells us to go ahead. He has taken in a first-class business man as partner,—one who has been engaged in manufacturing for many years, and who is thoroughly aroused to the fearful errors of the old system; and the firm has opened a fine, centrally located store, at No. 14 Union Square, New York. This change gives the enthusiastic inventor time and room for his great work. Heretofore, since we began to bring his grand improvement to the notice of our readers, he has hardly found time to eat or sleep. His days and evenings have chiefly been occupied in demonstrating his method to the multitudes who have flocked to him from all quarters, so that he could neither attend to his workmen nor his correspondence. The result is, that he has to-day about a bushel of unanswered letters, and is printing a circular to send in reply. This circular he accompanies with descriptive pamphlets, instructions for measuring, and price-list, such as he sends to all applicants by mail. With his new store, and his greatly increased facilities, he hopes to be able to do his duty by all in future.

It is not alone with suffering individuals that Mr. McComber has to deal. Our articles have so interested several of the most prominent manufacturers in the country, that they have called upon him, and have taken licenses to use the improved lasts. In fact, as we predicted three months ago, they have been fairly compelled to do this in self-defence, because intelligent people who have tried the new goods will not return to the old torturing affairs. Thus, Messrs. J. C. Bennett & Barnard, 51 Warren Street, New York, leading manufacturers of ladies' and children's shoes, employing more than two hundred workmen,—have determined to work in future on McComber's plan. Naturally enough, Mr. McComber looks upon the conversion of this very prominent house as a great achievement, and asks us to earnestly recommend them to the trade everywhere. This we gladly do, because we are conscious that we are doing a real service to the human race when we encourage the universal adoption of this blessed reform. Besides, we have taken pains to inform ourselves concerning the products of the factory of Messrs. J. C. Bennett & Barnard, and find the testimony in every way favorable. Their ladies' and children's boots and shoes have been classed among the best, both for the style and thoroughness of workmanship, and for the quality of the stock. Nothing, then, was needed but the adoption of a rational last, and their accession to the ranks of those who are fully converted to the religion of comfort in foot-gear, leaves nothing further to be demanded by us.

It stands to reason that this system should be used, to the exclusion of the absurd antediluvian mode, for it is at once elegant and comfortable. We have taken some pains to illustrate the subject, so as to fix our ideas more clearly in the minds of our readers. Here, for example, is the foot of a man of twenty-two, which has been abused in the good old way since he first began to wear shoes. There is nothing remarkable in it. There are millions more of the same sort of feet, all just as pretty as this. There are ladies, indeed, whose feet are far more ill-formed than this, because their feet are softer and more yielding, and therefore more readily respond to distorting pressure. The fortunate owner of this elegant foot has never tried to wear a shoe not large enough, but has been fitted by the ordinary system of measurement. The cut which we here show, exhibits the foot precisely as it stands. It was photographed, and this picture is an exact reproduction of the photograph. The boot which the gentleman was wearing was then cut open, so that the foot could be seen in its position, and another photograph was taken, which is shown above. The edge of the sole will be seen, with the upper leather bulging over it on both sides. The great-toe joint will be observed so far out of the position occupied by it in a natural state, that, if the distortion were suddenly induced, it would amount to absolute dislocation. This distortion has been occasioned by dragging the great-toe out of the true line, through the pressure of the upper leather upon it. In turn, the upper leather has been forced to thus drag the great toe toward the smaller ones by the pressure of the foot, not upon the sole but upon the upper, which presses outward far beyond the outline of the sole. The great strain is outwardly; for, although the distorted foot bulges out on the inner side, as shown in the above cuts, the bulging is induced by the extended joint, which forces a space for itself beyond the sole. In the case of a foot not seriously deformed, it will be found that the upper leather is forced beyond the line of the sole, outwardly only, while the upper of a shoe made on McComber's last, does not bulge over the sole at the ball, on either side, and can not do so.

Take now this cut. This is an exact view of the bottom of a boot not made on the McComber last, but probably as good a boot as can be found, constructed on the old plan. It had been worn long enough to take the form of the foot; to get what was called comfortable.

The boot was worn by Mr. Van Vleck, the engraver of these pictures, and was photographed on his foot. He had been wearing a pair of boots made by McComber, and saw that his own case afforded a fair illustration of the merits of the system. He accordingly had two photographs taken, the one above showing the ordinary boot in use, and another exhibiting the McComber boot on his foot, which we here present, and which we think shows clearly the symmetry and beauty of the new goods. There was never a boot made before the era of McComber, which, after being worn a couple of months, would not show a bulging of the upper leather over the sole.

Now, we wish to show views of the lasts, from different points. Here are top and bottom views. Here, also, are end views.

The idea will be given that the new last is crooked; that is, that it bends outwardly. But this is not true. It crooks as the foot does, nothing more. This is shown by the fact that any decent foot, not badly distorted, drops into it, and feels no pressure on any point; and that the foot, thus clothed, is perfectly fitted and looks well. A crooked look *does* accompany the last, if you look at the top of it; but this is because the ball, on the inside, overhangs the outline intended for the sole. If you look at the bottom of the last, or at the top of the completed boot, you will discover no appearance of crookedness. Here is an inside and an outside view of a McComber button-boot, photographed from two positions, on the foot of a lady; and we submit that it is a neat-looking structure. That it is comfortable we have ample testimony to prove. These boots have been worn for a couple of months, and have not given a moment's pain or uneasiness. Their wearer has been rather badly treated by shoemakers for the past thirty-two years,—as, in fact, have most people,—but her feet are now recovering from their deformity, and her comfort is complete. She is the wife of a leading writer for this journal, and gladly testifies to the solid comfort she enjoys in her McComber boots, on all occasions. She is anxious to tell the whole world what elegance and comfort the new system secures. The same feeling seems to pervade the minds of all who enjoy the blessed boon of comfortable feet. Crusty old fellows who have hobbled around for years with compressed toes, and called it gout or rheumatism, venting their irritation on everybody and everything, become kind and gentle as the pressure is removed, and comfort takes the place of torment. So, in an important sense, the inventor of this system is a missionary, preaching a gospel of beauty, of health, of comfort, and of freedom from deformity. As one estimable lady said to us, yesterday: "My temper and health have greatly improved since I began wearing McComber's shoes, and I bless him every day."

There is no doubt but that this noble improvement will become universal, and will serve to ameliorate some of the most fearful evils to which poor human nature is subjected. In this faith we are doing all we can, by tongue and pen, to encourage those who are laboring to extend the blessings of this reform. We want our intelligent readers to know what their duty is to themselves and to their children; and we hope to make it disgraceful to torture the human race in the future as it has been tortured in the past. If parents would but reflect upon the various effects of forcing the feet out of their normal shape, they would use every means within their knowledge to secure for their children freedom from the evils attending the ordinary methods of clothing the feet. They would seek fcr light among such as are competent to impart it; and, being convinced, they would guard their children from infancy to maturity against the fearful evils which ignorant fabricators of foot-clothing have for ages entailed upon the human race.

## Figures and Tables

**Figure f1:**
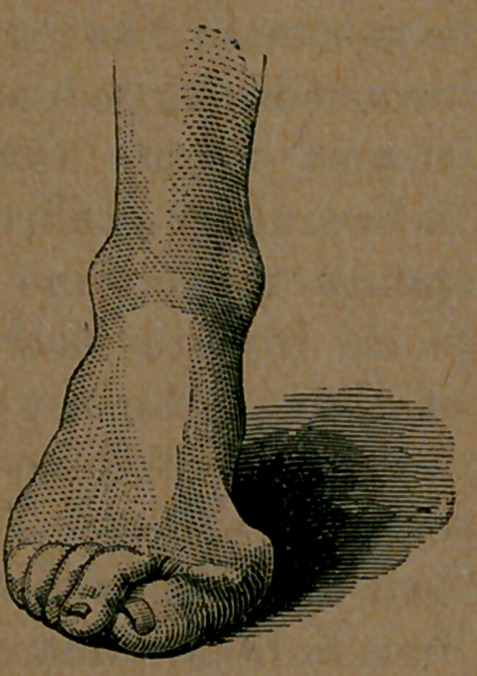


**Figure f2:**
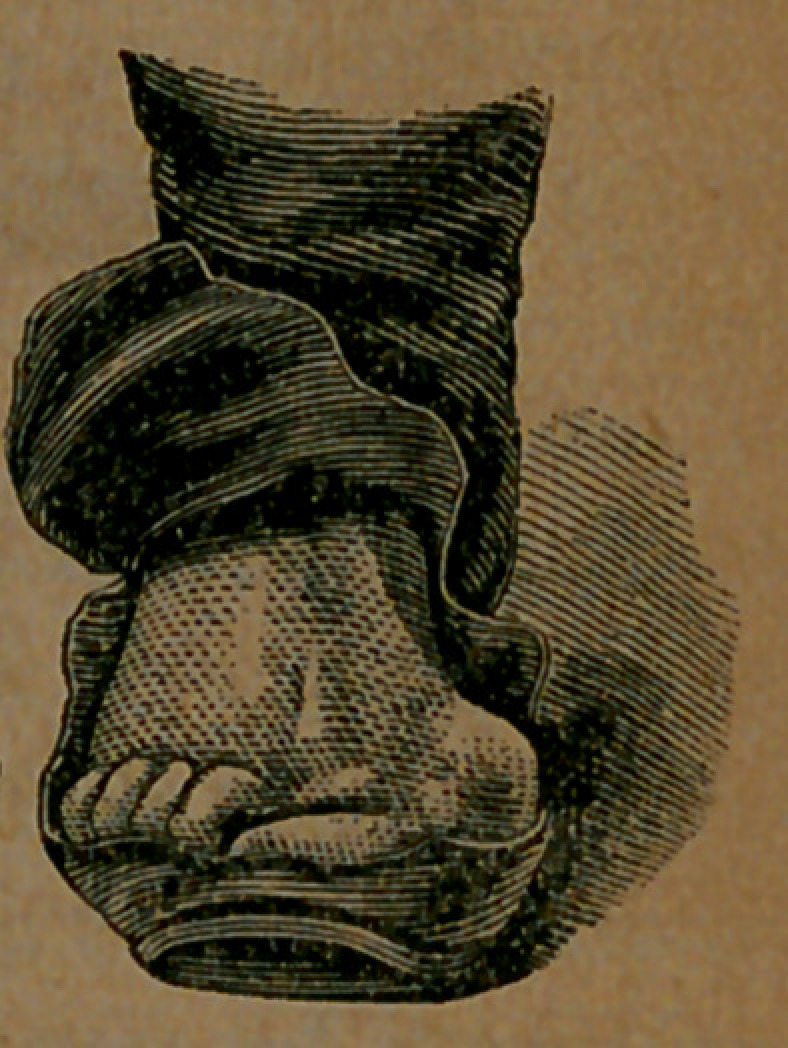


**Figure f3:**
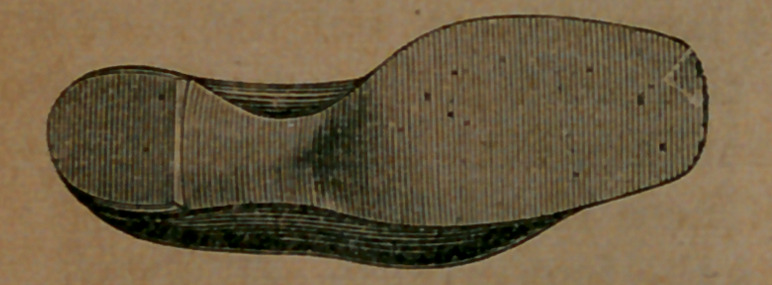


**Figure f4:**
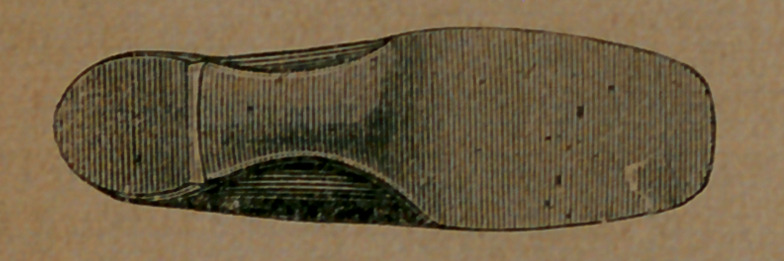


**Figure f5:**
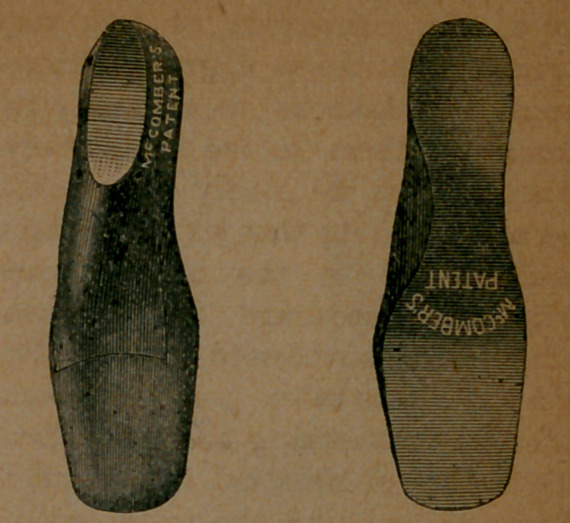


**Figure f6:**
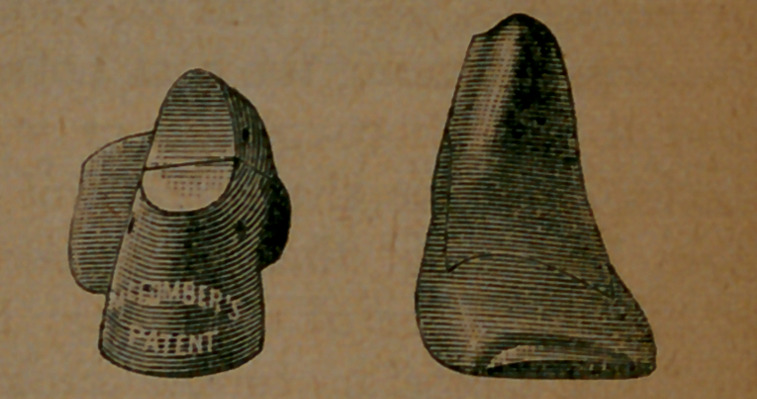


**Figure f7:**
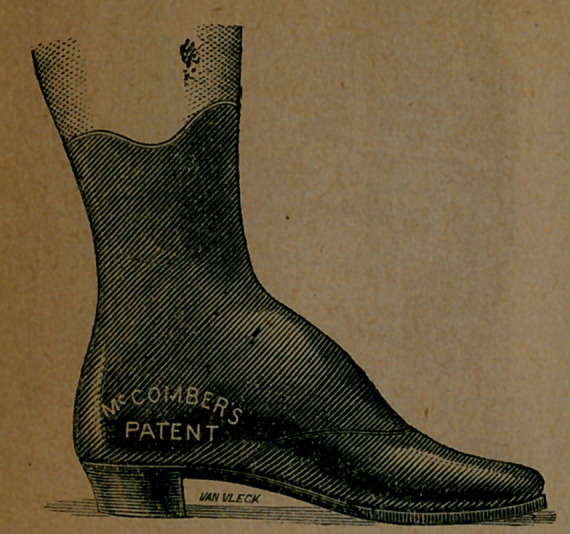


**Figure f8:**